# On the use of Mendelian randomization to assess the consequences of metformin exposure

**DOI:** 10.1093/ije/dyaa058

**Published:** 2020-05-07

**Authors:** Dylan M Williams

In a recent letter to the *IJE*, Zhou *et al.* attempt to assess whether metformin exposure would affect lung cancer incidence, using Mendelian randomization (MR).^1^ The authors should be commended for considering MR as a means to address this important question. MR may circumvent some of the biases inherent in conventional pharmacoepidemiology, such as confounding by indication, and thus has the potential to complement the evidence base on the issue. Zhou *et al.* conducted sound MR models to assess the effect of a long-term increase in circulating growth differentiation factor 15 (GDF15) on lung cancer. However, this is not equivalent to assessing the hypothesis that they proposed in their study. The adoption of circulating GDF15 as a proxy for metformin exposure is based on a fundamental misconception about how to instrument a drug exposure effectively with an MR design.

As the authors cite, an increase in circulating GDF15 is suggested to be a consequence of metformin use.^2^ This coincides with many other physiological responses to metformin treatment, including a marked decrease in blood glucose (one of the reasons for the drug being the first-line therapy for type 2 diabetes prevention and treatment). Individually, none of the responses following from drug use will fully encapsulate the effects of metformin exposure, and so none can be used in isolation to anticipate how the therapeutic will influence outcomes (see Figure 1 for an illustration of this issue). Rather, instrumenting variation in GDF15 as an exposure in MR models will assess whether the modulation of circulating GDF15 affects risk of an outcome. If the authors had reported an association of higher genetically-indexed GDF15 concentrations with lower risk of lung cancer, pharmacologists might have considered scouring our therapeutic armamentarium for agents to increase GDF15, and then to test any candidates as means to reduce lung cancer incidence. In such an instance, metformin would be implicated as a viable candidate for this purpose. This scenario shows how the use of MR to evaluate a biomarker’s causal relationship with an outcome can guide therapeutic discovery. In contrast, it is important to grasp that identifying a role (or lack thereof) of GDF15 in the aetiology of lung cancer does not necessarily offer any credible evidence to implicate or refute metformin’s ability to influence lung cancer risk, given that the drug could be having beneficial or detrimental effects on disease incidence by one or more distinct pathways.

**Figure 1:**
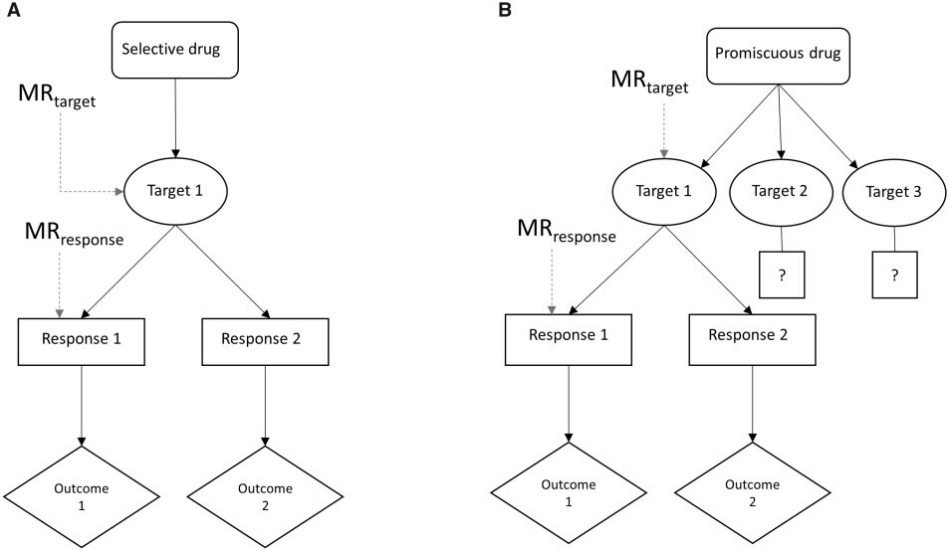
Instrumenting drug exposures effectively with Mendelian randomization. Panel A: for a selective drug with only one target, using a cis-MR design (MR_target_) to instrument the target’s function should model averaged effects via all pathways (responses 1 and 2) downstream of target modulation and predict consequences across these pathways (detecting effects on outcomes 1 and 2). In contrast, instrumenting a single downstream response of drug use (MR_response_), such as the change in circulating concentrations of a biomarker, will only predict consequences of this response (outcome 1) and not identify consequences incurred from effects of target modulation on other pathways (outcome 2). Panel B: for a promiscuous (non-specific) drug with multiple targets—as appears to be the case for metformin—a cis-MR design focusing on one of these targets may evaluate the averaged consequences of modulating this particular target, but will fail to predict the consequences of modulating other targets affected by exposure to the drug.

As analogy, consider statins in relation to coronary artery disease (CAD) prevention. In addition to reducing circulating low-density lipoprotein cholesterol—the major reason why these drugs prevent CAD events—statins also lower C-reactive protein (CRP), an inflammatory marker.^3^ If we were to instrument circulating CRP as a proxy for statin exposure in MR models as a means to evaluate whether statin use reduces CAD risk, we would not detect an effect because CRP does not appear to be a causal factor in CAD risk.^4^^,^^5^ Conversely, genetic variation in *HMGCR*, the gene which encodes the target of statins, is associated with CAD risk—implicating the enzyme targeted by statins in CAD aetiology.^6^ If we assess this variation in relation to CAD risk by its direction of effect on low-density lipoprotein cholesterol (LDL-C; variants which reduce LDL-C also lower the risk of CAD), we provide strong evidence to support the benefits of statins for CAD prevention. Here we should conclude that the use of variation at *HMGCR* can be informative in the repurposing potential, or pharmacovigilance for potential side effects, of exposure to statins or other drugs with similar mechanisms of action, whereas the use of genetically-indexed biomarkers of statin response for these purposes has more capacity to mislead us.

MR studies designed to address the effects of drug exposure on outcomes should, therefore, instrument the function of a drug’s target, not biomarkers related to drug use. Target-focused MR is more likely to encapsulate all of the (on-target) effects of drug exposure into an overall estimate, and hence predict the consequences of drug use more reliably. Instrumenting target function specifically has tended to involve the study of *cis*-acting variation in the vicinity of genes encoding a protein target of interest—an approach which has been termed *cis*-MR to mark the distinction between instrumenting drug target function and the instrumenting of any other traits, including biomarkers which may be downstream of drug exposure.^7^

Unfortunately, applying *cis*-MR to assess how metformin exposure may affect traits is likely to be prohibitively challenging, for at least three related reasons:

- considerable uncertainty about metformin’s target(s);
- possible non-selectivity, i.e. the drug may induce its effects via multiple targets;
- metformin may not exert its effects exclusively via proteins or other products encoded by the human genome.

Despite decades of study since the discovery of metformin, the drug’s target(s) are still uncertain. Part of the drug’s effects appear to be mediated by hepatic modulation of mitochondrial complex I and mitochondrial glycerol-3-phosphate dehydrogenase.^8^^,^^9^ However, a sizeable proportion of its glucose-lowering capacity may result from effects in the gastrointestinal tract, possibly via interactions with gut microbiota.^10^^,^^11^ Results from *cis*-MR to assess the efficacy of a drug (or surveillance for side effects) on the basis of uncertain targets should be interpreted very cautiously, and as an appraisal of the targets being addressed, rather than the consequences of using a related therapeutic. The possibility that metformin may be promiscuous and operate by more than one target also raises methodological questions, given that typical *cis*-MR designs assess the impact of modulating individual targets (a sound framework for addressing selective drugs designed for a single target) and address single proteins rather than complexes. Finally, when a drug’s target is not encoded by the human genome, the use of *cis*-MR for evaluating the drug’s effects may be precluded entirely, unless function of the non-human target could be instrumented satisfactorily by other data, e.g. bacterial genomics.

In conclusion, *cis-*MR is a valuable study design for addressing many questions in pharmacoepidemiology as well as drug discovery. Several methodological challenges are unique to these applications of MR, including different possibilities for variant selection criteria and various traits which might be used to index pharmacological action, e.g. variant associations with circulating concentrations of targets or downstream markers of target activity—areas of research where there is no current consensus.^12^ Paramount among these choices is the careful consideration of the target(s) of a given therapeutic, and whether we can claim to be indexing the anticipated effects of drug use reliably at all. Metformin is a cautionary case study in this respect.

## Funding

The author is funded by the UK’s Medical Research Council (MC_UU_00019/2).
